# Nanoparticle-Encapsulated Epirubicin Efficacy in the Inhibition of Growth of Orthotopic Ovarian Patient-Derived Xenograft in Immunocompromised Mice

**DOI:** 10.3390/ijms25010645

**Published:** 2024-01-04

**Authors:** Wioletta Kośnik, Hanna Sikorska, Adam Kiciak, Tomasz Ciach

**Affiliations:** 1 NanoVelos S.A., Rakowiecka 36, 02-532 Warsaw, Poland; 2 NanoGroup S.A., Rakowiecka 36, 02-532 Warsaw, Poland; 3 Faculty of Chemical and Process Engineering, Warsaw University of Technology, Waryńskiego 1, 00-645 Warsaw, Poland

**Keywords:** epirubicin hydrochloride, nanoparticles, patient-derived tumor xenografts (PDX), ovarian cancer, orthotopic ovarian cancer model

## Abstract

Epirubicin hydrochloride (EPI) is an anticancer drug widely used in the treatment of many solid tumors, including ovarian cancer. Because of its anatomical location, ovarian cancer shows symptoms when it is already in an advanced stage and is thus more difficult to treat. Epirubicin hydrochloride kills cancer cells effectively, but its dose escalation is limited by its severe toxicity. By encapsulating epirubicin in dextran-based nanoparticles (POLEPI), we expected to deliver higher and thus clinically more effective doses directly to tumors, where epirubicin would be released and retained longer in the tumor. The antitumor activity of POLEPI compared to EPI was first tested ex vivo in a series of ovarian cancer patient-derived tumor xenografts (PDX). The most promising PDX was then implanted orthotopically into immunocompromised mice, and tumor growth was monitored via magnetic resonance imaging (MRI). Although we succeeded in suppressing the growth of ovarian cancer derived from a patient, in a mouse model by 70% compared to 40% via EPI in 5 days after only one injection, we could not eliminate serious side effects, and the study was terminated prematurely for humane reasons.

## 1. Introduction

Ovarian cancer (OC) is the leading cause of death among all gynecological cancers in developed countries [1,2]. The standard treatment for women with OC involves aggressive debulking surgery and combination chemotherapy with a platinum compound and a taxane, resulting in complete clinical remission in up to 75% of cases, with a 5-year survival rate of ~30% [3,4]. Since OC develops deeply in the pelvic cavity, and clinical manifestations are limited in the early stage, most OC patients are diagnosed after significant disease progression has occurred. Moreover, owing to its high recurrence rate after resection and common chemoresistance, the mortality rate of OC is very high [5]. Although the use of anti-angiogenic agents and poly-ADP-ribose polymerase (PARP) inhibitors for maintenance therapy has increased median overall survival (OS) from 38.8 months to 51.7 months, tumors in most of treated patients will relapse and metastasize [6]. Moreover, relapsed patients develop drug resistance and suffer serious treatment side effects and eventually die, with the most common cause of death being intestinal obstruction. Different targeted therapies have been developed for OC, but the outcomes have not proved satisfactory. The new generation of immunotherapies has also been used to some extent for OC patients [7].

Toxicity is a major concern for anticancer drugs. These compounds present a narrow therapeutic index, with a small difference between the dose required for an antitumor effect and that responsible for unacceptable toxicity. Their recommended doses are determined according to the toxicity endpoint.

Anthracyclines have been in clinical practice since the 1960s and represent one of the most commonly used classes of anticancer drugs. EPI or pegylated liposomal doxorubicin (PLD) (Doxil(R)/Caelyx(R)) are the two most favorable anthracyclines in OC failing platinum-based chemotherapy [8,9,10]. EPI is very effective, but its accumulation in the human body can be highly toxic and could have severe adverse effects, such as cardiotoxicity, myelosuppression, and hypoalbuminemia, potentially causing irreversible damage to organs.

Targeting OC with nanoparticles containing EPI might allow for treatment with a high dose of EPI while reducing its toxicity. The physicochemical characteristics of nanomaterials such as size, charge, surface area, solubility, surface chemistry and shape influence their toxicity [11]. Immune stimulation, immune suppression and immune modulation have all been reported for nanomaterials, partly as a result of the formation of a protein corona made up of serum proteins [12,13,14]. In cancer applications, passive targeting is defined as the preferential accumulation of the drug in the tumor. The accumulation and delivery of the drug are determined by the capability of the drug delivery systems (DDSs) to overcome biological barriers and the inherent characteristics of the DDS itself [15,16]. Small molecule drugs can enter and exit tumors and normal tissues and typically do not accumulate in them. In contrast, nano-sized DDSs have been proven to be able to cause drug accumulation in tumors through exploiting the dimensions of fenestrae in tumor blood vessels and the lack of a proper lymphatic system [17,18]. 

In tumors and other proliferating or developing cells, the rate of glucose uptake dramatically increases and lactate is produced, even in the presence of oxygen and fully functioning mitochondria. This process is known as the Warburg effect [19,20]. The availability of glucose appears to be a result of direct competition between the tumor and tumor-infiltrating lymphocytes (TILs). There is direct evidence indicating that targeting aerobic glycolysis in the tumor has the added benefit of increasing the supply of glucose to TILs and thus boosting their main function, which is to eradicate the tumor cells [21]. 

Moreover, the rapid and high accumulation of polysaccharide-based nanoparticles, without specific homing molecules, may be due to the “per se” targeting of the saccharide coating to glucose transporters (GLUTs) in the cell membrane. The affinity of dextran-based nanocarriers for GLUTs has been described [22]. The overexpression of GLUTs in cancer cells results in an increased demand for glucose. 

On the other hand, increased glucose metabolism lowers the pH in the tumor microenvironment due to lactate secretion [23,24]. Exploiting the pH gradient between normal tissues and the tumor microenvironment to afford external drug release, and the low pH in cellular endosomes (~pH 5) to afford intracellular drug release, pH-sensitive nanoparticle systems have been designed to facilitate the release of anticancer drugs in a pH-controlled manner. The preparation and properties of doxorubicin encapsulated in nanoparticles have been described previously [25].

POLEPI is EPI encapsulated in nanoparticles (NPs). The finished product consists of EPI (active substance), polyaldehydedextran (PAD), dodecylamine hydrochloride (DDA) and alanine, all acting as NP-forming agents. The size distribution of POLEPI is within range of 88.6–150.4 nm. It has been shown in vitro in dialysis experiments that the release of EPI from POLEPI exhibited sustained and pH-dependent release profiles which is due to the pH-dependent bond between EPI and dextran nanoparticles that allows for the release of EPI with lowering pH (Supplementary Materials Table S1, Figures S1 and S2). 

Dextran nanoparticles may have advantages such as increased drug-loading capacity, enhanced penetration, improved cellular uptake, and thus increased local drug concentrations at the target tissue site [26]. The published research indicates that dextran nanoparticles can potentially have applications in the delivery of anti-tumor therapeutics with reduced off-site toxicity [27,28].

To test this hypothesis, we have administered POLEPI into immunocompromised mice bearing orthotopic epithelial ovarian (EO) patient-derived xenografts (PDXs). PDXs represent an evolution of the cell line xenograft model whereby fresh tumor tissue, obtained directly from patients, is implanted subcutaneously (s.c.), intraperitoneally (i.p.) or orthotopically into immunodeficient mice [29,30,31]. Each method has its own advantages and limitations for meeting specific needs. Prior to EOC (epithelial ovarian cancer) PDX implantation, we have evaluated ex vivo the cytotoxicity of POLEPI and compared it to free EPI (EPI) and nanoparticle (NP) blank cytotoxicity in a series of PDX models representing human EOC to select the most promising model to investigate the advantage of POLEPI vs. EPI as an anti-EOC therapeutic agent. 

Our preliminary results indicate that although POLEPI was superior to EPI in terms of tumor volume reduction, it exhibited unexpected off-target toxicity in this orthotopic EOC PDX model.

## 2. Results

### 2.1. Ex Vivo Cytotoxicity of POLEPI in Champions TumorGraft^®^ and Certis Oncology Solutions PDX Models

The primary purpose of these assays was to ensure that there was a dose response in a given PDX model to EPI and POLEPI and to select the best PDX based on the IC_50_ values, tumor take rate and growth rate in order to move forward with the in vivo studies in mice bearing the selected PDX, implanted either subcutaneously or orthotopically, to compare the anti-tumor activity of POLEPI to EPI. 

Half maximal inhibitory concentration, or IC_50_, is a measurement representing the halfway point in which a compound of interest produces the complete inhibition of a biological or biochemical function. IC_50_ is most often used as a measurement of antagonist or inhibitory drug potency, as well as a quantification of the toxicological effects of inhibitory compounds.

**Figure 1:**
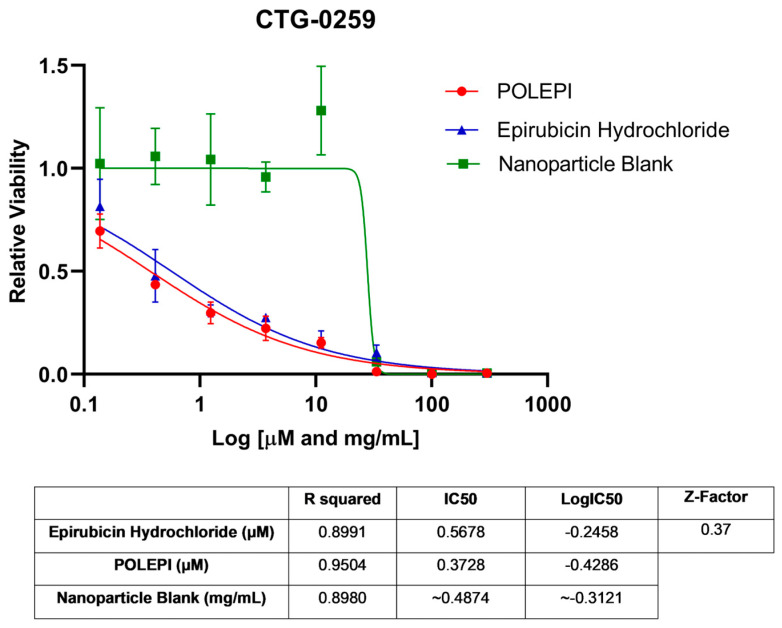
Ovarian cancer model CTG-0259 test agents’ dose response.

**Figure 2:**
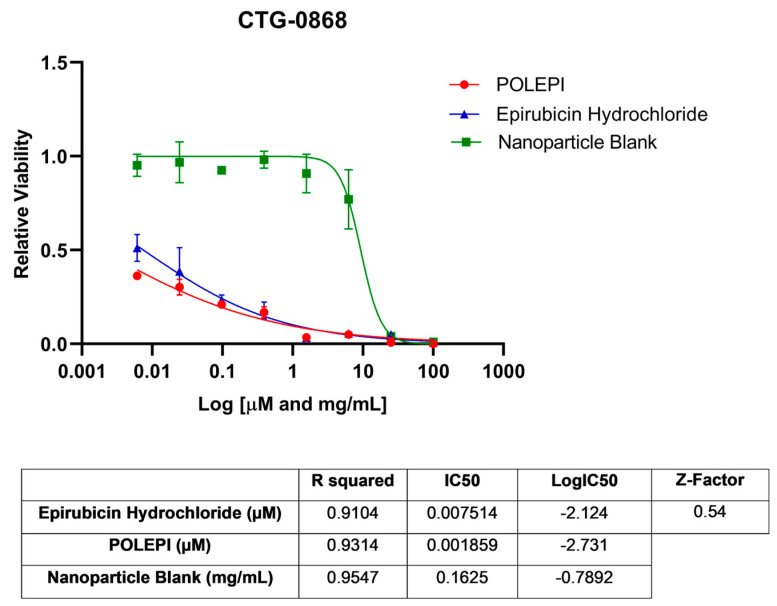
Ovarian cancer model CTG-0868 test agents’ dose response.

**Figure 3:**
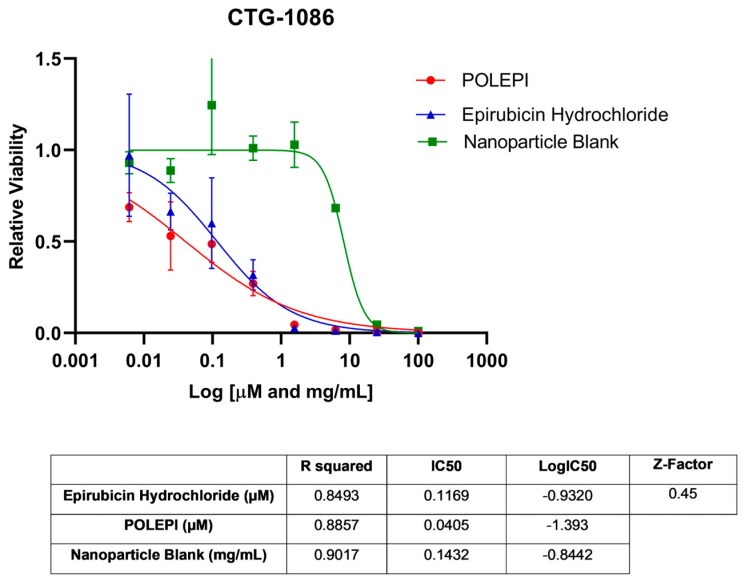
Ovarian cancer model CTG-1086 test agents’ dose response.

First, POLEPI and EPI were evaluated in two Champions TumorGraft^®^ models (Rockville, MD, USA), CTG-0259 and CTG-0964, in eight concentrations ranging from 0.1 to 300 µM and NP Blank from 0.002 to 5.24 mg/mL. Since the pre-selected dose levels were too high, CTG-0868, CTG-0964 (a repeat that failed quality control QC) and CTG-1086 were tested in eight new concentrations ranging from 0.006 to 100 µM EPI/POLEPI and NP Blank from 0.00011 to 1.75 mg/mL. Table 1 summarizes the calculated IC_50_ values and QC data. Dose-response graphs are presented in Figure 1, Figure 2 and Figure 3. The CTG-0964 model failed QC again and the graph is not presented here. Champions TumorGraft^®^ model CTG-0259 was selected for subcutaneous implantation into athymic Nude-Foxn1^nu^ female mice, but its take rate during propagation and growth rate were too slow to be acceptable for an in vivo efficacy study, and thus this model was discontinued. 

**Figure 4:**
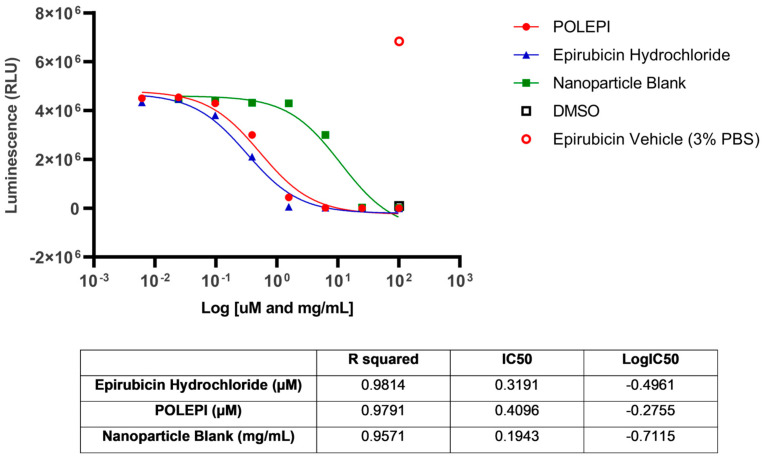
Ovarian cancer model CRT_OV_00367 test agents dose-response.

The assay conducted at Certis Oncology demonstrated that model CRT_OV_00367 was sensitive to treatment with both EPI and POLEPI ex vivo. NP Blank required a much higher dose to see toxicity. The calculated IC_50_ values were 0.3191 µM for EPI, 0.4096 µM for POLEPI and 0.1943 mg/mL for NP Blank (Figure 4). This tumor take rate was good, and thus CRT_OV_00367 was selected for orthotopic implantation at Certis Oncology Solutions.

### 2.2. In Vivo Determination of MTD of POLEPI and EPI in Non-Tumor-Bearing Immunocompromised Mice

**Figure 5:**
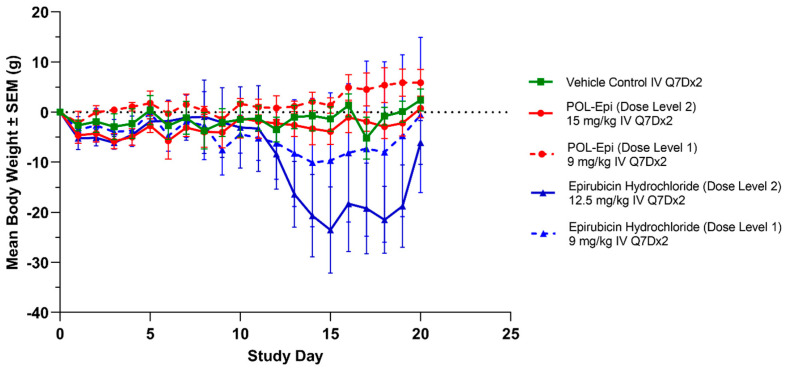
Mean percent body weight changes over time in non-tumor-bearing immunocompromised mice.

In the first tolerability study, Q7D×2 IV (intravenous) treatment of mice with vehicle control, NP Blank at 602 mg/kg, and POLEPI at 12.5 mg/kg were tolerated. Treatment with EPI at 20 mg/kg and POLEPI at doses of 20 mg/kg or 30 mg/kg was not tolerated due to acute body weight loss resulting in many unscheduled deaths or euthanasia (Supplementary Materials Table S4 and Figure S3). In part II, Q7D×2 IV treatment with EPI at 9 mg/kg and POLEPI at doses of 9 mg/kg or 15 mg/kg were tolerated. EPI at 12.5 mg/kg was not tolerated (Figure 5). Thus, MTD for EPI was established to be 9 mg/kg and for POLEPI, 15 mg/kg.

### 2.3. In Vivo Efficacy of POLEPI in Immunocompromised Mice Bearing Orthotopic Ovarian PDX

**Figure 6:**
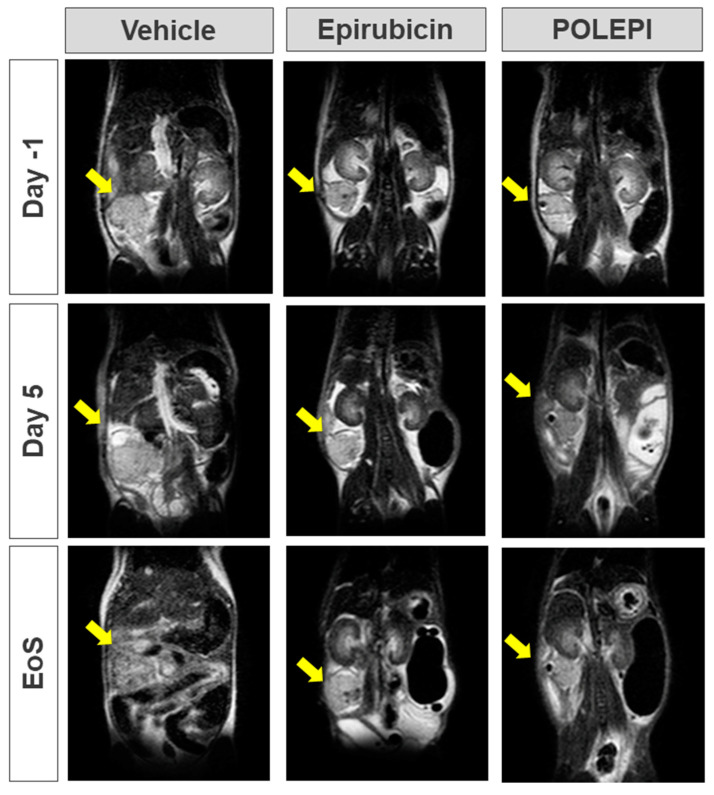
Orthotopic PDX tumor in Certis Orthotopic ovarian model visualized via MRI. Yellow arrow indicates tumor. EoS: Day 7–9; vehicle: AN#001 for Day-1, Day 5 and EoS; epirubicin: AN#022 for Day-1, Day 5 and EoS; and POLEPI: AN#011 for Day-1, Day 5 and EoS.

The objective of this study was to determine the therapeutic efficacy of POLEPI at the MTD of 15 mg/kg compared to the EPI MTD of 9 mg/kg by measuring tumor volumes via MRI. Once the efficacy was established, a full dose response efficacy study followed. Unexpectedly, multiple animals in EPI group 2 and POLEPI group 3 had to be removed from the study earlier due to animal body weight loss of >20% or death. The study was prematurely terminated on Day 9 and none of the animals received the second dose. Tumor volumes were determined on Day-1, and the subsequent MRI images were taken on Study Day 5 and then prior to animal termination, whenever possible. The final MRI images of the tumors in group 1 were taken on Study Day 9 (Figure 6). 

**Figure 7:**
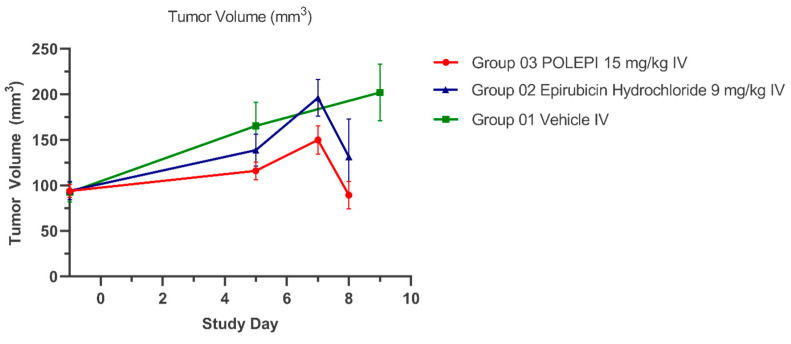
Average Tumor Volumes (mm^3^) Over Time.

**Figure 8:**
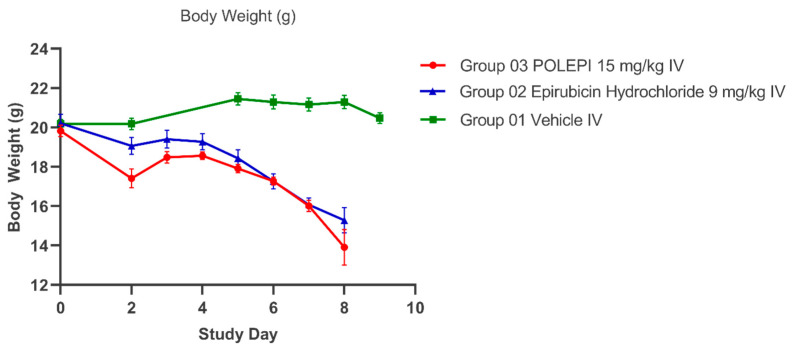
Average body weight (g) over time.

The average tumor volumes are presented in Figure 7 and the individual animals in Table 2. The average body weight change (%) over time is presented in Figure 8.

In order to compare the antitumor efficacy of POLEPI vs. EPI, tumor growth inhibition % (TGI%) and tumor doubling time (DT) were calculated as efficacy parameters for each treatment group (T) versus the control (C) using initial (i) and final (f) tumor volume measurements according to the following equations: %TGI = 1 − (Tf − Ti)/(Cf − Ci) × 100, where for this study, Day 0 was actually Day-1 (randomization) and the final day of taking tumor measurements was actually Day 5 on which the majority of animals were still alive.

As can be seen in Table 2, a single administration of POLEPI caused, by Day 5, tumor reduction by 70% compared to 39% as caused by EPI (difference not statistically significant).

Tumor growth rate (TGR) is an early indicator of antitumor drug activity in phase I clinical trials [32]. Tumor doubling time was determined for each treated group using the formula DT = (Df − Di) × log2/(logTVf − logTVi), where D = Day and TV = Tumor Volume, assuming a constant growth [33].

As can be seen in Table 2, a single administration of POLEPI increased the tumor doubling time from 6 days in the NP Blank control group to 16 days, thus significantly slowing down tumor growth, compared to the doubling time of 9 days caused by EPI.

## 3. Discussion

The antitumor activity of POLEPI compared to EPI could not be reliably evaluated in this orthotopic ovarian PDX model as treatment with both POLEPI and EPI led to severe body weight loss, deaths and early study termination. However, a single administration of POLEPI caused, on Day 5, tumor reduction by 70% compared to 39% as caused by EPI (difference not statistically significant) and increased tumor doubling time from 6 days in the NP Blank control group to 16 days, thus significantly slowing down tumor growth.

In the clinic, EPI as a single agent for OC treatment can be used at doses of 50–90 mg/m^2^, which corresponds to 16.6–30 mg/kg in mice, at 3–4-week intervals for several cycles until a cumulative dose of 900 mg/m^2^ is reached. It is recommended by the EMA/FDA that the starting dose for the first-in-human (FIH) study is safe but, at the same, as close as possible to being pharmacodynamically effective. Thus, the proposed dose level of POLEPI for this pharmacodynamic (PD) study was 15 m/kg, the MTD.

The severe toxicity of both EPI and POLEPI (due to the EPI content and not the nanoparticle matrix) was unexpected in view of both our preceding studies and the published literature. Chida T et al. demonstrated, in BALB/C nu/nu mice with orthotopic breast cancer (MDA-MB-231-luc-D3H2LN tumor cell line), the therapeutic effect of EPI-loaded polymeric micelles (EPI/m), administered at 10 or 20 mg/kg, with no significant body weight loss [34]. EPI at the MTD (10 mg/kg) in their study also showed an equivalent antitumor effect. Furthermore, they revealed that the improvement of the activated drug distribution of EPI/m contributed to a dose-dependent enhancement of the antitumor effect through the expansion of the therapeutic window. Takahashi et al. developed NC-6300 that comprised EPI covalently bound to PEG polyaspartate block copolymer through an acid–labile hydrazone bond [35]. The conjugate forms a micellar structure of 40–80 nm in diameter in an aqueous milieu. NC-6300 (10, 15 mg/kg) and EPI (10 mg/kg) were given IV three times to BALB/c nude mice (CLEA) bearing s.c. or an orthotopic liver xenograft of human hepatocellular carcinoma Hep3B/Luc cells. There was a significant difference between the control group and the groups treated with EPI and NC-6300. A comparison of the relative photon count revealed significant differences between NC-6300 at 15 mg/kg and EPI at 10 mg/kg. The Kaplan–Meier analysis showed that there was a significant improvement in the survival rate in the group given NC-6300 at 10 mg and 15 mg/kg compared with that in the group given EPI at 10 mg/kg. No deaths were reported by Day 40. The MTD in nude mice administered with NC-6300 and native EPI, three times with a 4-day interval between each dose, were 25 and 9 mg/kg, respectively [36].

However, similar to our observations, unexpected anticancer drug toxicity in severely immunocompromised mice was reported by others. Maletzki C. et al. engrafted four different individual colorectal (CRC) PDX models into NOD.Cg-PrkdcscidIl2rgtm1Wjl (NSG) mice and treated them with a cytostatic drug, 5-fluoruracil (5-FU) [37]. The engraftment efficacy was high as expected in NSG mice, yielding a stable PDX growth for therapy stratification. However, the overall tolerability towards 5-FU was unexpectedly low, whereas the prodrug capecitabine as well as the combination of 5-FU/LV at low doses were well tolerated. In their another (unpublished) study, doxorubicin given at a very low dose to leukemic NSG mice (PDX of different acute lymphoblastic leukemia) resulted in all mice being deceased within seven days of therapy (single or double i.p. application of 8 and 4 mg/kg, respectively). With these results, they propose the hypothesis that metabolisms are quite individual among laboratory animals and drug sensitivities are mouse-strain specific. Thus, in addition to the necessity of complex immunodeficiency, variations in pharmacogenetics among mice strains should be taken into consideration. Though not systematically addressed in the literature, there is evidence of differential tolerability among individual mouse strains towards certain cytostatic drugs. Antitumor drug toxicity in tumor-free and tumor-bearing mice differ. The effect of the disease on standard endpoints of drug toxicity were studied [38], e.g., it has been reported that pharmacokinetics of therapeutic antibodies depend on the mice strain used [39].

PDXs are the most clinically relevant models currently used in the EOC field [40]. Orthotopic xenograft models are free from the multiple constraints of subcutaneous xenograft models [41]. It has recently been documented that mice implanted with PDX fragments largely retained the genetics of the human tumors from which they were initially created [42]. Nutrients and signaling provided by the mouse ovary microenvironment allow for tumor development in conditions similar to those in EOC patients. As such, orthotopic xenograft models are an important step in preclinical studies [43]. However, despite their notable advantages, these models are technically challenging, which limits their application in EOC research. Correlations between the drug response in patients and PDXs derived from the same patients before treatment have been observed in several studies [44,45]. 

PDX clinical trials (PCTs) are significant for clinical decision making before human clinical trials and the development of anti-cancer agents. PCT is referred to as “phase II type clinical trial-like models”. In 2015, Gao et al. designed a high-throughput in vivo drug screening method, “1  ×  1  ×  1”, which outlined one animal per model per treatment using a large number of PDX models [46]. 

It is possible that the complex immunodeficiency reduces tolerance to EPI, thus making those mice especially sensitive. The CIEA (Central Institute for Experimental Animals in Japan) NOG mouse^®^ from Taconic (NOD.Cg-Prkdcscid Il2rgtm1Sug/JicTac) used in our efficacy study is a severely immunodeficient model lacking mature T, B, and NK cells, with reduced complement activity, dysfunctional macrophages and dendritic cells, but it is superior for the engraftment of human cells and tissues. For the MTD determination, we used immunocompromised athymic Nude-Foxn1^nu^ mice from Envigo (Indianapolis, Indiana). These mice have a normal B-cell function but are T-cell deficient (no generation of cytotoxic effector cells and no graft versus host response) and have dysfunctional rudimentary thymus. We do not know at present whether the observed increased toxicity to EPI in our model is due to severe immunodeficiency, the species used, changed EPI metabolism, or the effect of the disease on the sensitivity of normal tissues to EPI or how relevant it could be for the determination of the starting dose for the Phase 1 clinical trial, including predicting human efficacious doses. Our observations might be particularly pertinent for designs of PCT trials, and maybe performing a dose-finding pre-study in more than one immunocompromised mouse species with different drug regimens might be warranted, especially, in view of the recently reported data from the statistical analyses of preclinical toxicities and clinical toxicities from published Phase 1 trials for 108 drugs, showing that animal models did not accurately predict the toxicity profile of the drugs in humans [47].

## 4. Materials and Methods

### 4.1. Materials

Test Articles: Epirubicin HCl (Ellence^®^, NDC 0009-5091-01, Pharmacia & Upjohn Company LLC, New York, NY, USA) manufactured by Pfizer Injectables was supplied as the sterile solution at a concentration of 2 mg/mL (3.45 mM) (calculated as EPI HCl content). POLEPI and NP Blank were supplied by NanoVelos Sp. z o.o., Warsaw, Poland as lyophilized powders in glass vials. The POLEPI vial contained 3.32% (33.532 mg) EPI in a total of 1.0065–1.01 g powder. NP Blank contained 1.21–1.23 g powder (PAD+ DDA+ alanine).

#### Test Articles Stock and Working Solution Preparation

Each agent’s stock solution was prepared fresh at a suitable concentration to perform consistent dilutions for each replicate well. POLEPI was reconstituted with PBS pH 7.4 to prepare a working stock solution at a concentration of 2 mg/mL (3.45 mM) (calculated as epirubicin HCl content). NP Blank was reconstituted with PBS pH 7.4 to prepare a stock solution of 60.24 mg/mL.

Initially, higher concentrations of EPI stocks were prepared. Aliquots of POLEPI, EPI and NP Blank stocks were diluted with 1X PBS or sterile water (WFI) (for EPI) to generate 300 μM and 5.24 mg/mL working stock solutions (the target step was a 3-fold dilution). Furthermore, 300 μM and 5.24 mg/mL stocks were used to achieve dilution points 0.1–300 μM of EPI and POLEPI and NP Blank from 0.002 to 5.24 mg/mL in eight concentrations. Appropriate volumes of each stock solution were added to test wells. For subsequent studies, 100 μM and 1.75 mg/mL stocks were used to achieve dilution points 0.006–100 μM for EPI and POLEPI and NP Blank from 0.00011 to 1.75 mg/mL in eight concentrations (a 4-fold dilution).

### 4.2. Ex Vivo Evaluation of POLEPI and EPI Using Low Passage Champions TumorGraft^®^ Models

These studies were carried out by Champions Oncology, Inc., Rockville, MD, USA using four of their OC PDX models, with the anticipation of using one of these models for subcutaneous implantation into immunocompromised mice for POLEPI efficacy testing.

Model CTG-0259 was derived from a 71-year-old patient with recurrent metastatic disease stage III, histologically defined as a poorly differentiated papillary carcinoma of serous origin. The patient previously responded to liposomal doxorubicin treatment.

Model CTG-0964 was derived from a 56-year-old patient with recurrent metastatic disease stage IV, histologically defined as a poorly differentiated papillary carcinoma of serous origin. The patient has never been treated with anthracyclines.

Model CTG-0868 was derived from a 72-year-old patient with recurrent metastatic disease stage IV, histologically defined as a poorly differentiated endometrial carcinoma, a papillary carcinoma of serous origin. The patient previously did not respond to liposomal doxorubicin treatment. 

Model CTG-1086 was derived from a 65-year-old patient with recurrent metastatic disease stage III, histologically defined as a poorly differentiated papillary carcinoma of serous origin. The patient has never been treated with anthracyclines.

#### 4.2.1. Tumor Fragment Assay for the Determination of IC_50_ Values

Freshly thawed PDX fragments were engrafted into a single animal and then sequentially passaged into more mice. The stock animals used in this study were athymic Nude-Foxn1nu (immune-compromised)/NOG female mice 6–8 weeks of age obtained from Athymic Nude-Envigo/NOG-Taconic.

On Day 0, warm tumors between approximately 500 and 1500 mm^3^ were excised and placed into MACS^®^ media (Miltenyi Biotec, Inc., Auburn, CA, USA) with anti–anti (antibiotic + antimycotic) and stored on wet ice until dissociated into fragments using enzymatic digestion. Large fragments were removed by filtration (using a 500 μm filter followed by a 200 μm filter). Once filtered, fragments from each model were suspended in PDX media (DMEM high glucose + 10% FBS + anti–anti + 1:500 Primocin antimicrobial agent + 1:100 Glutamax) and seeded at a density of 1000 cells/20 μL per well into 384-well plates for CellTiter-Glo^®^ (Promega, Madison, WI, USA) analysis. The CellTiter-Glo^®^ luminescent cell viability assay is a homogeneous method used to determine the number of viable cells in culture based on the quantitation of the ATP present, which signals the presence of metabolically active cells. Media were plated in the perimeter wells to avoid edge-effect artifacts. The plates were kept in a 37 °C/5% CO_2_ incubator. The media were not changed during the 6-day incubation period. Tumor fragments were treated with 20 μL of 2× EPI, POLEPI, and NP Blank at the appropriate concentrations directly dispensed into wells on the next day (Day 1) using DragonFly Discovery (SPT LabTech, Covina, CA, USA) The total volume was 40 μL/well. Each group had quadruplicate wells, including 10% DMSO as a positive control, an untreated control with PDX media as background for Z-factor calculation, 10% PBS or 3% PBS, and 10% or 3% WFI were used as the negative controls. Additionally, 4 wells with PDX fragments were left untreated. At the study endpoint, Day 6, the plates were removed from the incubator and equilibrated to room temperature for up to 30 min. Furthermoer, 40 μL of CellTiter-Glo^®^ was added to wells and mixed for 5 min at high speed and 25 min at low speed on a plate shaker. The luminescence was recorded using a Spectramax plate reader (Molecular Devices, LLC., San Jose, CA, USA). 

#### 4.2.2. Data Analysis and Efficacy Evaluation 

A relative luminescence unit (RLU) was used to measure viable cells. RLU values for every test well were corrected for the background. The efficacy was evaluated by calculating IC_50_ values using GraphPad Prism 9.0.0 software to generate the IC_50_ curves. Cell viability (*y*-axis) was plotted against drug concentrations (*x*-axis in μM or mg/mL) using a linear–log-formatted semi-log plot, and a sigmoid curve was generated by nonlinear regression of the data (a four-parameter logistic model with a minimum constraint of 0 and a maximum constraint of 1). Dose-response inhibition (log (inhibition)) vs. normalized response—variable slope resulted in the generation of IC_50_. Log IC_50_, and R2 values were used to determine the goodness of the fit. For Z-factor, to ensure that the comparison of the negative control to the background was acceptable, positive control values had to be discernably lower than negative control values.

### 4.3. Ex Vivo Evaluation of POLEPI Using Certis Oncology Ovarian PDX for Orthotopic Engrafting

CRT_OV_00367 is serous ovarian cancer obtained from the bowel metastasis of a female patient that received prior treatment with carboplatin, paclitaxel, doxorubicin, olaparib, topotecan, gemcitabine, and bevacizumab. Three female NOG mice were implanted with the CRT_OV_00367 tumor subcutaneously. The mice were monitored until the animals reached a tumor volume of 500–1000 mm^3^. Tumor tissue was collected, dissociated, and then plated in a 96-well format, in 50 µL of media/well. Furthermore, 20% DMSO in media was prepared for a 10% DMSO final concentration (positive control) and EPI, POLEPI, and NP Blank were prepared at a 2× concentration for subsequent serial dilution (4-fold and 8-dose points) to achieve an EPI concentration range of 100–0.006 µM, POLEPI 77.24–0.004714 µM, and NP Blank 1.75–0.00043 mg/mL (7-dose points). 50 µL of test agents and DMSO were added to the respective cell containing wells for a 1x final concentration and then incubated for 4 days at 37 °C in a humidified CO_2_ incubator. For the bioluminescence analysis of ATP content/viable cells to determine the IC_50_, the plates and CellTiter-Glo^®^ (Promega, Madison, WI, USA) were equilibrated for 30 min at RT in the dark, 100 µL/well of the CellTiter-Glo^®^ was added at RT, and Varioskan^®^ Lux (Model #N7-00020) using SkanIt^®^ 6.0 software (Thermo Fisher Scientific, Waltham, MA, USA) was run.

### 4.4. In Vivo Determination of Maximum Tolerated Dose (MTD) in Non-Tumor-Bearing Immunocompromised Mice

These non-GLP studies were conducted in two parts at Champions Oncology, Inc., Rockville, MD, USA. The initial starting dose range for POLEPI was based on the results of previously conducted studies at the Medical University of Białystok, Poland, in which it was determined that the MTD for POLEPI was less than 31.5 mg/kg and more than 24.23 mg/kg in healthy non-immunocompromised mice (Supplementary Materials Tables S2 and S3). 

In Part 1, 18 athymic Nude-Foxn1^nu^ immune-compromised (Envigo, Indianapolis, IN, USA) non-tumor-bearing female mice (n = 3/group) received intravenously (IV) two doses, 7 days apart, of WFI as the vehicle control, NP Blank at 602 mg/kg, EPI at 20 mg/kg, or POLEPI at doses of 12.5 mg/kg, 20 mg/kg, and 30 mg/kg. In Part 2, 15 athymic nude-Foxn1nu mice were randomized into 5 groups (n = 3) that received IV WFI, EPI at doses of 9 mg/kg and 12.5 mg/kg, or POLEPI at doses of 9 mg/kg and 15 mg/kg. In both parts, the first doses were administered on Day 0 and the second ones on Day 6. Animals were weighed and monitored daily. On dosing days, animals were weighed prior to dose administrations. Animals exhibiting ≥10% net body weight loss when compared to Day 0 were provided with DietGel^®^ 76A (ClearH2O^®^, Westbrook, ME, USA) ad libitum. The study was concluded on Day 20. 

Tolerability was assessed according to body weight loss, lethality, and clinical signs of adverse treatment-related side effects. Data, including individual and mean gram weights, the mean percent weight change versus Day 0 (%vD0), were recorded for each group and %vD0 plotted at study completion. The final weight was taken on the day the study reached the endpoint or if the animal was found moribund, if possible. Animal deaths were recorded. Groups reporting a mean loss of %vD0 >20 and/or >10% mortality were considered above the MTD for that treatment on the evaluated regimen. Additional toxicity endpoints for the study were any animal exhibiting >20% net weight loss for a period lasting 7 days or if the mice displayed >30% net weight loss when compared to Day 0 which were considered moribund and humanely euthanized. The maximum mean %vD0 (weight nadir: the lowest weight recorded during the study from Day 0) for each treatment group was reported at study completion.

Descriptive statistical analysis was performed using GraphPad Prism 9.0.0.

### 4.5. In Vivo Evaluation of POLEPI in a Certis Oncology Solutions Orthotopic CRT_OV_00367 Model in Immunocompromised Mice

In total, 45 female NOG (Taconic, Germantown, NY, USA) mice were implanted with CRT_OV_00367 tumor fragments orthotopically. The mice were anesthetized using isoflurane induction, shaved and surgically prepped using a surgical scrub and 70% isopropyl alcohol. An incision was made to skin below the animal’s right rib cage and was gently blunt dissected over the peritoneal wall. An incision was made to the peritoneal wall over the right ovary, and it was gently removed from the cavity. A tumor fragment was sutured onto the ovary. The peritoneum was sealed with sutures, and wound clips were used to close the skin. Analgesics were administered. Interrupted sutures were placed to the incision site. A post-operative analgesic was administered. Animals were imaged weekly using the Aspect Imaging M3 MRI to track tumor growth. The tumor volume was quantified using VivoQuant 2021 software (Invicro, Needham, MA, USA). Imaging was also performed prior to sacrificing the animals when possible.

When the tumors reached a range of 55–162 mm^3^ with an average of 94 mm^3^, 30 mice were randomized to the respective treatment groups and dosed within 72 h. Day-1 is defined by randomization, and Day 0 is defined by dosing start. Group 1 (n = 10) received NP Blank, Group 2 EPI (Ellence, NDC 0009-5091-01, Pharmacia & Upjohn Company LLC, New York, NY, USA) at 9 mg/kg, and Group 3 POLEPI at 15 mg/kg. All injections were carried out via IV into the tail vein. Initially, all animals were to receive test articles weekly for 4 weeks. Clinical observations were performed twice weekly. The animals were checked for any signs of behavioral abnormalities, and clinical signs were recorded as observed. Body weights were measured twice weekly following the randomization and initiation of treatment. Starting on Study Day 2, body weights were taken daily for Group 2 EPI and Group 3 POLEPI due to body weight loss concerns. Starting on Study Day 5, body weights were taken daily for all animals, including Group 1 NP Blank. Body weight loss was calculated based on the body weight of the mouse on the first day of treatment (Study Day 0). Starting on Study Day 2, Diet Gel was given to all animals, as well as twice daily saline injections. Only one dose of a test agent was given on Study Day 0, and dosing holidays were given to all animals thereafter due to severe body weight loss. In total, 14 animals were euthanized when a body weight loss of >20% was observed.

## 5. Conclusions

POLEPI effectively suppressed tumor growth by Day 5 and reduced the tumor doubling time 2.5-fold following a single IV administration. However, the off-target toxicity remained very high. POLEPI is able to deliver more drugs, although the efficacy of POLEPI compared to epirubicin was not fully evaluated due to the treatment-derived body weight loss in this model. The question of whether lower doses are as effective but less toxic remains to be seen. Are severely immunocompromised mice bearing PDX the right model to test new anticancer drugs to predict the response and dose for Phase 1 clinical trials?

## Figures and Tables

**Table 1:** Summary of IC_50_ values of EPI, POLEPI, and NP Blank in Champions TumorGraft Models ex vivo.

Model ID	Z-Factor	Test Agent	IC_50_	R^2^	Log IC_50_
CTG-0259	0.37	Epirubicin Hydrochloride	0.5678 µM	0.8991	−0.2458
		POLEPI	0.3728 µM	0.9504	−0.4286
		Nanoparticle Blank	~0.4874 mg/mL	0.8980	~−0.3121
CTG-0868	0.54	Epirubicin Hydrochloride	0.007514 µM	0.9104	−2.124
		POLEPI	0.001859 µM	0.9314	−2.731
		Nanoparticle Blank	0.1625 mg/mL	0.9547	−0.7892
CTG-0964	0.33	Epirubicin Hydrochloride	0.008680 µM	0.7393	−2.061
		POLEPI	0.006624 µM	0.7399	−2.179
		Nanoparticle Blank	0.1324 mg/mL	0.8442	−0.8780
CTG-0964 *	−0.45	Epirubicin Hydrochloride	35,175,064 µM	0.03104	7.546
		POLEPI	2.806 × 10^17^ µM	0.001213	17.45
		Nanoparticle Blank	~0.3647 mg/mL	0.4510	~−0.4381
CTG-1086	0.45	Epirubicin Hydrochloride	0.1169 µM	0.8493	−0.9320
		POLEPI	0.04049 µM	0.8857	−1.393
		Nanoparticle Blank	0.1432 mg/mL	0.9017	−0.8442

Note: where R^2^ values are below 0.8, IC_50_ values cannot accurately be calculated. Multiple outliers were omitted from the dose curve. An acceptable Z factor must lie between the range of 0-1-1: ideal, 0.5–1: excellent assay, 0–0.5: marginal Assay, and <0: unreliable assay. Model CTG-0868: in the first attempt, this model barely passed internal QC (Z factor = 0.1) and was repeated. The repeat assay passed QC (Z factor = 0.5) and is depicted in this table. Model CTG-0964: the assay was successful, but IC_50_ values for EPI and POLEPI could not be accurately calculated (R^2^ < 0.8). The dose level was reduced, and the repeat assay for this model was performed using the highest dose of 100 µM. This repeat assay with lower doses failed internal QC as indicated below in CTG-0964 *. * Model CTG-0964 (repeat, failed QC): the repeated attempt with this model failed QC (Z factor < 1).

**Table 2:** Tumor growth inhibition % (TGI%) and tumor doubling time (DT).

Group	Animal ID	Absolute MRI Tumor Volume (mm³)						TGI%	TDT
		Study Days							
		−1 (0)	5	7	8	9	Day 5–Day 0		
Group 1: NP Blank	001	127.32	296.41			340.08	169.09		
Group 1: NP Blank	002	128.30	254.47			340.25	126.17		
Group 1: NP Blank	008	108.23	201.34			256.85	93.11		
Group 1: NP Blank	009	58.64	91.05			93.84	32.41		
Group 1: NP Blank	014	54.03	76.73			76.82	22.70		
Group 1: NP Blank	015	89.65	153.55			183.41	63.90		
Group 1: NP Blank	034	57.00	74.51			132.83	17.51		
Group 1: NP Blank	037	100.67	186.78			209.81	86.11		
Group 1: NP Blank	039	143.44	235.30			273.96	91.86		
Group 1: NP Blank	040	57.16	83.97			112.02	26.81		
Average		92.44	165.41			201.99	72.97		5.96
Group 2: EPI	004	161.61	147.71	159.14			−13.90		
Group 2: EPI	005	67.52	105.44				37.92		
Group 2: EPI	007	103.96	140.56	163.01			36.60		
Group 2: EPI	017	90.06	144.50		172.88		54.44		
Group 2: EPI	022	61.85	87.43		89.73		25.58		
Group 2: EPI	028	69.25	66.78				−2.47		
Group 2: EPI	036	91.62	179.38				87.76		
Group 2: EPI	038	133.24	241.22	233.00			107.98		
Group 2: EPI	043	88.17	197.96	229.05			109.79		
Group 2: EPI	045	73.86	75.91				2.05		
Average		94.11	138.69	196.05	131.31		44.58	38.91	8.94
Group 3: POLEPI	006	105.52	121.64				16.12		
Group 3: POLEPI	011	54.12	74.02		58.56		19.90		
Group 3: POLEPI	018	97.21	159.39				62.18		
Group 3: POLEPI	026	72.79							
Group 3: POLEPI	029	104.20							
Group 3: POLEPI	032	77.39	108.97		102.72		31.58		
Group 3: POLEPI	033	90.06	102.23		106.26		12.17		
Group 3: POLEPI	035	93.76	141.13	156.84			47.37		
Group 3: POLEPI	041	141.54	124.93	172.55			−16.61		
Group 3: POLEPI	044	101.49	95.08	120.49			−6.41		
Average		93.81	115.92	149.96	89.18		22.12	69.69	16.37

TGI% = 1 − (Tday5 − Tday0)/(Cday5 − Cday0) × 100; TDT = (day5 − day0) × log(2)/(logTVday 5 − logTVday 0).

## Data Availability

The data presented in this study are available upon request.

## Supplementary Materials

The supporting information can be downloaded at: https://www.mdpi.com/article/10.3390/ijms25010645/s1. References [25,48,49,50,51] are cited in the supplementary materials.
