# Supplementary material for: Nanoparticle-Encapsulated Epirubicin Efficacy in the Inhibition of Growth of Orthotopic Ovarian Patient-Derived Xenograft in Immunocompromised Mice
Source: Int J Mol Sci. 2024 Jan 4;25(1):645. doi: 10.3390/ijms25010645 (PMC10779551; doi:10.3390/ijms25010645)
Supplement: Supplementary file 1 [file ijms-25-00645-s001.zip › ijms-2789478-supplementary.pdf]

Supporting information

Nanoparticle-Encapsulated Epirubicin Efficacy in the Inhibition of Growth of Orthotopic Ovarian Patient- Derived Xenograft in Immunocompromised Mice

Wioletta Kośnik [1,*], Hanna Sikorska [1], Adam Kiciak [2] and Tomasz Ciach [1,2,3,*]

[1] NanoVelos S.A, Rakowiecka 36, 02-532 Warsaw, Poland

[2] NanoGroup S.A., Rakowiecka 36, 02-532 Warsaw, Poland

[3] Faculty of Chemical and Process Engineering, Warsaw University of Technology, Waryńskiego 1, 00-645 Warsaw, Poland

* Correspondence: w.kosnik@nanogroup.eu (W.K.); tomasz.ciach@pw.edu.pl (T.C.)

1. Synthesis 1
   1.1. Synthesis of polyaldehydodextran 1
   1.2. Synthesis of dextran nanoparticles with epirubicin (EPI-NPs) 2
   1.3. Synthesis of empty dextran nanoparticles 2
   1.4. Determination of epirubicin hydrochloride concentration in NV-EPI-222 based on calibration curve and by means of UV-Vis spectroscopy 2
   1.5. Procedure of preparation of stock solution of EPI HCl (epirubicin hydrochloride) in NV-EPI-222 (POLEPI) 3
   1.6. Procedure of preparation of stock solution of NV-carrier-221 4
2. In vitro drug release 4
3. Nanoparticles diameter and size distribution 5
4. Evaluation of the maximum tolerable dose (MTD) of EPI-NPs (POLEPI) 6
   4.1. Mice body weight—study T425/001/2017 6
   4.2. Mice body weight—study T425/002/2017 6
   4.3. Results—study T425/001/2017 7
   4.4. Results—study T425/002/2017 9
5. Tolerability Study I—Champions Oncology 10

1. Synthesis

1.1. Synthesis of polyaldehydodextran.

The dextran (70 kDa, pharmacopeia grade, Pharmacosmos, Holbaek, Denmark) was oxidized according to a modified protocol of Muangsiri and Fuentes [48,49]. Briefly, 25.5 g of dextran was dissolved in 500 mL ultra-pure water (Type 1; conductivity < 1 μS/cm at 25 °C, resistivity 18 MΩ-cm at 25 °C). Subsequently, 6.73 g of sodium metaperiodate (Sigma-Aldrich) was added in molar ratios of 1:10 (IO_4^-/glucose units) to obtain approximately 14% (13,78%) of glucose ring oxidation. The solution was stirred in the dark at room temperature for 1 h. The post-reaction solution was purified with distilled water using a tangential flow filtration (GAMBRO, Polyflux 14L). After two hours of filtration, the titration

method (iodide and iodate method) was used to confirm the absence of iodates in the wastewater. The final product was freeze-dried or spray-dried which allowed the product to be obtained in the form of a white solid. The number of aldehyde groups in polyaldehydodextran (PAD) was determined using a modified hydroxylamine hydrochloride method [50] with unmodified dextran as a reference. The final product was stored in the refrigerator in a sealed container.

1.2. Synthesis of dextran nanoparticles with epirubicin (EPI-NPs).

Dextran nanoparticles with epirubicin were prepared according to the preparation method of nanoparticles from polysaccharides as described in the earlier publication [25] using dextran with a molecular weight of 70 kDa (oxidation degree ~14%) and dodecylamine hydrochloride as a coiling agent.

The following outlines the procedure for the synthesis of dextran nanoparticles with epirubicin: 1.5 g of dried PAD (polyaldehyde dextran) was dissolved in 15 ml of ultra-pure water (Type 1; conductivity < 0.1 μS/cm at 25 °C, resistivity 18 MΩ-cm at 25 °C) at 30 °C, and 15.0 mL of 1% solution of epirubicin hydrochloride was added. Then, 4.044 mL of 2% solution of dodecylamine hydrochloride was added. Before using them, all solutions of the reagents were warmed to 30 °C. The mixture was constantly stirred at 30 °C for 30 min (magnetic stirrer, 350 rpm). The pH was measured and increased with a 0.1 M sodium hydroxide solution within 60 min until the solution reached pH 9. At this time, 4.314 mL of 4% solution of alanine was added. Finally, the pH of the mixture was decreased to 7.4 with 0.1 M HCl solution. The final product was lyophilized which allowed the product to be obtained in the form of an orange-red solid.

EPI-NPs was prepared with an average size of 100-200 nm as measured in aqueous solutions using NanoSight LM 10 (405 nm laser). The determined epirubicin content in a dry matter of nanoparticles was approximately 4%. The obtained nanoparticles were freeze-dried and stored in sealed containers at a temperature of 2-8 °C.

1.3. Synthesis of empty dextran nanoparticles

The following outlines the procedure for the synthesis of dextran nanoparticles: 6.4 g of dried PAD was dissolved in 64 mL of ultra-pure water, and 16.7 mL of 2% solution of dodecylamine hydrochloride was added. Before using them, all solutions of the reagents were warmed to 30 °C. The mixture was constantly stirred at 30 °C for 30 min. The pH was measured and increased with a 0.5 M sodium hydroxide solution within 60 min until the solution reached pH 9. At this time, 19.0 mL of 4% solution of alanine was added. Finally, the pH of the mixture was decreased to 7.4 with 0.5M HCl solution. The final product was lyophilized. NPs were prepared with an average size of 137.2 ± 4.3 nm as measured in aqueous solutions using NTA and DLS. The obtained nanoparticles were freeze-dried and stored in sealed containers at a temperature of 4 °C.

1.4. Determination of epirubicin hydrochloride concentration in NV-EPI-222 based on calibration curve and by means of UV-Vis spectroscopy

Three random stock solutions of NV_EPI_222 were prepared in pre-warm and sterile PBS. Samples were prepared to contain at maximum 7.0 mg of a nanocarrier. Each of three samples was dissolved in 1 ml of sterile and pre-warm PBS. In order to dissolve the compound completely, the solutions were mixed for ca. 30 min. From each prepared stock solution, a portion of 100 μL was collected and dissolved in 2.9 mL of PBS in order to obtain a dilution appropriate for UV-Vis measurements. Next, for each final sample, UV-Vis absorption spectra were measured in the range 200-1000nm in quartz cuvettes with a cell path length of 1cm. The absorption band at λmax = 480nm was used

as the reference one for the calculation of the EPI HCl concentration.

The mass and percentage contribution of epirubicin hydrochloride in NV-EPI-222 samples are as follows:

Sample number	NV-EPI-222 weight [mg]	Abs at λ_{max}= 480nm (solution: 100µL stock sample + 2.9 ml of PBS)	EPI HCl concentration in measured sample [mM]	EPI HCl concentration in stock sample [mM]	EPI HCl weight [mg]	**% EPI HCl in NV-EPI-222**
1	3.2	0.077	$6.39*10^{-3}$	0.1917	0.111	**3.47%**
2	6.5	0.134	$1.20*10^{-2}$	0.2089	0.209	**3.21%**
3	4.8	0.104	$9.05*10^{-3}$	0.2715	0.157	**3.28%**

1.5. Procedure of preparation of stock solution of EPI HCl (epirubicin hydrochloride) in NV-EPI-222 (POLEPI)

NV-EPI-222: each vial contains 1.01 g of POLEPI with 3.32% of EPI HCl what gives 33.53 mg of the active compound. PBS (calcium and magnesium-free for cell culture) for NV-EPI-222 dissolution

Parameter	Value	Unit
**EPI HCl molecular weight [g/mol]**	580	g/mol
**NV-EPI-222 sample weight [g]**	1.01	g
**% Epi HCl**	3.32%	
**EPI HCL weight in NV-EPI-222 [g]**	0.033532	g
**EPI HCL weight in NV-EPI-222 [mg]**	33.532	mg
**Stock concentration of EPI HCl in NV-EPI-222 [mg/mL]**	2	mg/mL
**Stock concentration of NV-EPI-222 [mg/mL]**	60.24	mg/mL
**Volume of PBS for NV-EPI-222 dissolution [mL]**	16.766	mL
**Volume of PBS for NV-EPI-222 dissolution [µL]**	16766	µL
**Volume of PBS for NV-EPI-222 dissolution [L]**	0.016766	L
**Mol number of EPI HCl in NV-EPI-222 [mol]**	5.78138E-05	mol
**Stock solution of EPI HCl (in NV-EPI-222) [mol/L]**	0.00345	mol/L = M
**Stock solution of EPI HCl (in NV-EPI-222) [mmol/:]**	3.45	mmol/L = mM

Furthermore, 1.01 g (1 vial) of NV-EPI-222 was dissolved in 16 mL and 766 µL of sterile and pre-warm PBS to obtain 2 mg/mL (3.45 mM) stock solution of Epi HCl captured in NV-EPI-221 (60.24 mg/mL POLEPI solution). The stock solution was then used to prepare a series of dilutions. For the dilutions, a cell medium was used. The type of cell medium was chosen based on the type of cell culture that was involved in ex vivo studies.

1.6. Procedure of preparation of Stock Solution of NV-carrier-221

NV-carrier-221: each vial contains 1.23g of blank nanoparticle

PBS (calcium and magnesium-free for cell culture) for NV-carrier-221 dissolution

Parameter	Value	Unit
**NV-carrier-221 sample weight [g]**	1.23	g
**NV-carrier-221 sample weight [mg]**	1230	mg
**Stock concentration of NV-carrier-221 [mg/mL]**	60.24	mg/mL
**Volume of PBS for NV-carrier-221 dissolution [mL]**	20.418	mL
**Volume of PBS for NV-carrier-221 dissolution [µL]**	20418	µL

Moreover, 1.23 g (1 vial) of NV-carrier-221 was dissolved in 20 mL and 418 µL of sterile and pre-warm PBS to obtain 60.24 mg/mL stock solution. The stock solution was then used to prepare a series of dilutions. For the dilutions, a cell medium was used. The type of cell medium was chosen based on the type of cell culture that was involved in ex vivo studies.

2. *In Vitro* drug release

The release of epirubicin from the NPs was evaluated using the dialysis method (DM) [51]. The EPI-NPs were investigated under physiological conditions (PBS, pH 7.4) and simulated cancerous conditions (PBS, pH 5.5) in vitro over a 48-hour period. Freeze-dried drug-loaded NPs were suspended in water for self-assembly (final drug concentration 1 mg/mL) under gentle stirring for 30 min. As a control of the epirubicin release rate from the dialysis bag, a water solution of epirubicin was used (EPI concentration 1 mg/ml). Ten mL of the suspension was then placed inside the dialysis bag (Carl Roth, MWCO 12 – 14 kDa) in glass flasks that contained 100 ml of phosphate-buffered saline (PBS), pH 7.4 and 5.5, as release media. The samples were kept at 37°C and were light-protected (laboratory shaker). The release of epirubicin from EPI-NPs is higher in tumor-simulating conditions due to lower pH. This is due to the pH-dependent bond between epirubicin and dextran nanoparticles that allows for the release of epirubicin with lowering pH.

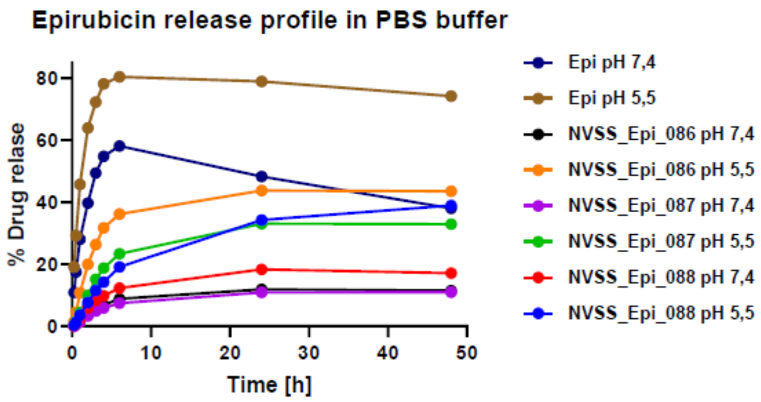

Figure S1. Epirubicin release profile determined by UV-Vis spectrophotometer.

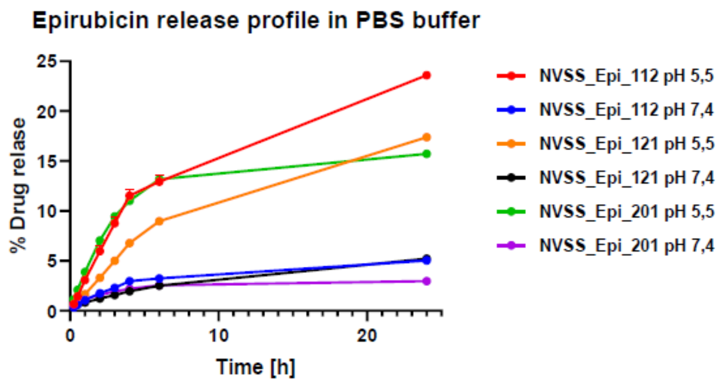

Figure S2. Epirubicin release profile determined by reversed-phase high-performance liquid chromatography (RP- HPLC).

3. Nanoparticles diameter and size distribution

The particle size and size distribution of NPs and POLEPI were determined using an LM 10 HS NanoSight instrument (Malvern Instruments Ltd.) with 405 nm laser scattering and Nanoparticles Tracking Analysis software. The measurements were replicated at least three times. The particle size of NPs was within the range 107.3-137.2 nm and the particle size of POLEPI was within the range 88.6 -150.4 nm (Table S1).

Table S1. Dextran NPs and POLEPI particle size

Sample / batch name	Particle size (nm)	Standard deviation (±nm)
**Dextran nanoparticles (NPs)**		
NVSS111_nośnik[a]	107.3	4.6
NVSS_Nośnik_219B	137.2	4.3
**Final product POLEPI**		
NVSS112_EPI[a,c]	123.3	4.8
NVSS121_EPI	88.6	4.8
NVSS_EPI_201	117.5	5.8
NVSS_EPI_207	139.4	17.7
NVSS_EPI_209	150.4	10.6
NV_SS_EPI_219[b]	131.8	4.1
[a] lyophilized without cryoprotectant<br>[b] product without dialysis after synthesis<br>[c] zeta potential -3.13mV		

4. Evaluation of the maximum tolerable dose (MTD) of EPI-NPs (POLEPI).

The study was conducted in accordance with the Organisation for Economic Cooperation and Development (OECD) Guidelines 425/EU B.7 and in accordance with Good Laboratory Practice (GLP). The study was approved by the Local Ethics Committee for Animal Experiments in Olsztyn (Resolution No. 18/2017 of 28.02.2017).

The project was coordinated by the Medical University of Białystok with Dr. hab. n. med. Małgorzata Michalina Brzóska, head of study T425/001/2017 and T425/002/2017.

The toxicity of acute dextran nanoparticles POLEPI (EPI-NPs) was assessed using the higher and lower dose method according to OECD procedure No. 425/EU B.7 with modifications appropriate for the purpose of this study.

4.1. Mice body weight—study T425/001/2017

The results of the measurements of the body weight of female mice during the 14-day observation period after the administration of the test material are presented in Table 2. The body weight of mice after 14 days after the administration of EPI-NPs and NPs remained practically unchanged or increased slightly (by about 1 - 2 g).

Table S2. Body weight of the female mice (BALB/ccmdb-strain mice aged 9 - 10) during the experiment.

Test material	Dose (mg/kg BW)[c]	Mouse number	Body weight (g)			
			Initial weight	Weight after the 1st week	Weight after the 2nd week	Change in BW during the observation period
NPs	100	1	21.50	21.20	21.00	−0.50
	320	6	21.20	20.20	20.55	−0.65
	1024	11	20.20	20.50	20.80	0.60
	3277	17	20.25	21.15	22.20	1.95
EPI-NPs	31.5	4	22.10	23.55	---*	---*
	40.95	9	21.60	---**	---**	---**
	31.5	14<br>15	21.15<br>21.00	21.60<br>21.10	---*<br>---*	---*<br>---*
	24.23	20<br>23<br>24	21.15<br>20.20<br>20.45	21.70<br>21.30<br>20.60	20.90<br>20.60<br>21.20	- 0.25<br>0.40<br>0.75

[c] doses of EPI-NPs were given in terms of EPI respectively.
* - the mouse was humanely euthanized on the 7th day after EPI-NPs were administered.
** - the mouse was humanely euthanized about 9 hours after EPI-NPs were administered. *

4.2. Mice body weight—study T425/002/2017

The body weight of mice after 14 days of EPI-NPs administration (Table 3) remained virtually unchanged or slightly increased (by about 0.40 - 1.70 g) or decreased (by about 0.05 - 0.80 g). The body weight of mice that received the free EPI drug decreased significantly (by about 2.10 - 3.95 g). All mice in this group (EPI) were skinny and were euthanized for humane reasons on Day 4 at both the 30 and 31 mg/kg doses.

Table S3. Body weight of the female mice during the experiment.

Test material	Dose (mg/kg BW) [c]	Mouse number	Body weight (g)			
			Initial weight	Weight after 1st week	Weight after 2nd week	Change in BW during the observation period
EPI-NPs	27.39	1	20.50	19.10	20.10	−0.40
	28.64	2	20.90	20.95	22.60	1.70
	30.00	3	21.00	19.30	19.90	−0.10
	30.00	5	20.45	19.40	20.40	−0.05
	30.00	6	20.30	19.80	20.70	0.40
	31.00	7	20.30	19.80	20.70	0.40
	31.00	8	20.30	20.70	19.50	−0.80
	31.00	9	20.80	20.80	21.70	0.90
EPI	30	10**	20.40	17.90	------	−2.50***
	30	11**	20.60	18.50	------	−2.10***
	30	12**	20.65	18.50	------	−2.15***
	31	13**	20.85	16.90	------	−3.95***
	31	14**	20.85	18.10	------	−2.75***
	31	15**	20.50	16.70	------	−3.80***

[c] doses of EPI-NPs were given in terms of EPI respectively.
** - the mouse was humanely euthanized on the 4th day after EPI was administered.
*** - weight change within 4 days of administration of EPI (mouse was humanely euthanized on the 4th day after administration).

4.3. Results – study T425/001/2017

The study started with an evaluation of the acute toxicity and MTD of EPI-NPs and NPs (study code T425/001/2017). The initial dose of EPI-NPs contained the equivalent of a median lethal dose of EPI for mice with intravenous administration (LD_{50} = 31.5 mg EPI/kg bodyweight), and the factor by which the dose was being modified was equal to 1.3. EPI-NPs were administered to seven mice in total in the following dosages (all doses of EPI-NPs were administered as equivalents of EPI): 24.23 mg/kg BW (three mice), 31.5 mg/kg BW (three mice), and 40.95 mg/kg BW (one mouse). The initial dose of NPs was equal to 100 mg/kg BW and was being modified according to OECD No. 425 by a factor of 3.2. NPs were administered to four mice in total in the following dosages: 100, 320, 1024, and 3277 mg/kg BW.

The administration of NPs in the dosages of 100, 320, and 1024 mg/kg BW to mice did not result in the occurrence of any symptoms of toxicity noticeable during general and detailed observation in the first day after administration or during the 14-day period of observation. In the mouse to which the NPs were administered in a dosage of 3277 mg/kg BW, immediately after administration, a slumped posture, fur standing on end, decreased physical activity, cutaneous muscle spasms, and dyspnea were observed. The majority of the changes mentioned above were mild and faded during several hours since administration. These changes were not observed already after about 4 h after administration. Moreover, in this mouse, no further abnormalities were noticed during general and specific observations in the course of the subsequent (14-day) observation. None of the mice died during the 14 days after the administration of NPs. In the mice to which NPs were administered, pathological changes mainly in the liver (vacuolar degeneration of various severity), kidneys (low severity of changes), and lungs (blood stasis or hyperaemia) were noticed. Histopathological changes in the mice to which the NPs were administered in the dosages of 100, 320, and 1024 mg/kg BW, did not differ in character, although an insignificant increase in their magnitude, depending on dosage, was observed. In the liver, the kidneys, and the lungs of the mice to which NPs were administered in a dosage of 3277 mg/kg BW, the histopathological changes were more severe than with lower dosages. The changes were very severe, possibly leading to cell death and organ dysfunction. The body weight of mice during the 14 days after the administration of NPs stayed virtually constant or increased insignificantly (by about 2 g maximum). NPs in the dosage of 3277 mg/kg BW did not result in any deaths or severe toxicity noticeable during the clinical observation in the period of 14 days after the administration and in the macroscopic examination during the autopsy, but due to the inability of administering a higher dosage of NPs to the mice (lack of solubility of higher amounts of NPs in the small amount of water for injection), subsequent dosages comprising the increase of the 3277 mg/kg BW dosage by a factor of 3.2 were not administered. Based on the results of the performed studies, it can be inferred that the MTD (defined as a dose effecting toxicity symptoms but not a significant reduction in the survival time of the animals) of NPs (with intravenous administration) is higher than 3277 mg/kg BW.

The mouse to which EPI-NPs were administered in the dosage of 40.95 mg/kg BW was humanely killed after 9 h from administration due to the occurrence of severe symptoms of toxicity. All three mice to which EPI-NPs were administered in the dosage of 31.4 mg/kg BW survived for 48 h from administration. In two mice, light pink tinting of urine was observed and in one, a mild and brief decrease in physical activity directly after administration. Severe symptoms of toxicity in all mice occurred on the 7th day from administration (decrease in physical activity, ascites, mild dehydration, vocalization at an attempt of palpations of the abdomen). Due to the occurrence of these symptoms, all three mice were humanely killed on the 7th day since the administration of EPI-NPs in the dosage of 31.5 mg/kg BW. In the mice to which EPI-NPs were administered in the dosage of 24.23 mg/kg BW outside of the change of urine tint, no further abnormalities on the 1st day and during the 14-day observation were noticed. None of the three mice died during the 14 days since the administration of EPI-NPs at this dosage, and their body weight stayed virtually constant. The histopathology of the internal organs of two of the mice to which EPI-NPs were administered in the dosage of 31.5 mg/kg BW was significantly changed in comparison with the third mouse. In two animals, significant pathological changes were noticed, whereas the organs of the last mouse were very insignificantly changed morphologically or their image was normal. In all mice, to which EPI-NPs were administered in the dosage of 24.23 mg/kg BW, morphological changes to the organs (with the exclusion of the jejunum) were severe.It was necessary to humane kill the mice approximately 9 h after the administration of EPI-NPs in the dosage of 40.95 mg/kg BW. The occurrence of severe toxic effects in all three mice administered with EPI-NPs at a dose of 31.5 mg/kg BW also made it necessary to humanely kill the mice on the 7th day after administration. There was also an absence of deaths and toxic effects noticeable within

14 days after administration and the absence of significant abnormalities on macroscopic examination in the three mice administered with EPI-NPs at a dose of 24.23 mg/kg BW. Considering that the LD50 of EPI (when administered intravenously) is 31.5 mg/kg BW, it can be concluded from the results of the study that the encapsulation of EPI in dextran nanoparticles reduces the toxicity of the drug.

4.4. Results—study T425/002/2017

Since, throughout the course of study T425/001/2017, the MTD of EPI-NPs could not be precisely established due to the dose modification factor (1.3) turning out to be too high (it was proved that the dosage is lower than 31.5 mg/kg BW and higher than 24.23 mg/kg BW), further studies aimed at precisely ascertaining the dose were planned (study T425/002/2017). In study T425/001/2017, the three mice to which EPI-NPs were administered in the dosage of 31.5 mg/kg BW survived for 48 h after the administration, but after a week, they were killed due to humanitarian causes because of the occurrence of severe symptoms of toxicity. In the three mice to which EPI-NPs were administered in the dosage lowered by the factor of 1.3, i.e., 24.23 mg/kg BW, both during the 48 h after administration and in the 14-day observation period, no symptoms of toxicity were observed. Due to the above circumstances, the studies were continued with a lower dose modification factor (in the range between more than 1 and less than 1.3). The initial dose of EPI-NPs contained the equivalent of 27.39 mg EPI/kg BW (one mouse), and the initial factor was equal to 1.15. EPI-NPs were administered to eight mice in total in the following dosages: 27.39 mg/kg BW (one mouse), 28.64 mg/kg BW (one mouse), 30 mg/kg BW (three mice), and 31 mg/kg BW (three mice). EPI, as reference material for EPI-NPs, was administered in the dosages of 30 and 31 mg/kg BW (three mice for every dosage).

The results of clinical observations made on the 1st day and during the 14-day observation after the administration of EPI-NPs to three mice in the dosage equivalent to 30 mg EPI/kg BW and the results of macroscopic and histopathological examinations of the internal organs of these animals indicate that the MTD of EPI-NPs (with intravenous administration) is equal to 30 mg/kg BW (as equivalent to EPI).

The fact that all mice to which EPI was administered in the dosage of 30 mg/kg BW (three animals), and 31 mg/kg BW (three animals) were killed humanely on the 4th day after the administration of the drug, due to the occurrence of toxicity symptoms, in conjunction with the results of macroscopic and histopathological studies of the internal organs of these animals, and in comparison with the results of analogous studies on mice to which equivalent dosages of the drug in the formulation with dextran nanoparticles (EPI-NPs) were administered, allows us to infer that the encapsulation of the drug in dextran nanoparticles lowers the toxicity of the drug. All mice to which EPI-NPs in the dosage equivalent to 30 mg EPI/kg BW (three animals) and 31 mg/kg BW (three animals) were administered, survived for 14 days after the administration, and during the 14-day clinical observation, no abnormalities were observed in these animals outside of a transient change in urine color to light pink for a brief period after administration. All mice to which EPI was administered in the dosage of 30 mg/kg BW (three animals) and 31 mg/kg BW (three animals), in turn, were autopsied already on the 4th day after the administration, due to the occurrence of severe toxicity symptoms. Pathological changes in macro- and microscopic images of the internal organs were more severe in mice to which EPI was administered than in animals to which an equivalent dosage of a drug conjugated with dextran nanoparticles (EPI-NPs) was administered.

5. Tolerability Study I—Champions Oncology

Table S4. Study design for tolerability study I in non-tumor-bearing immunocompromised mice.

Group	n	Agent	Dose (mg/kg)	Dose Volume (mL/kg)	Route	Dosing Schedule	Total # of Doses
1	3	Vehicle	-	10	IV	Q7Dx2	2
2	3	Control Nanoparticle	602	10	IV	Q7Dx2	2
3	3	Epirubicin Hydrochloride	20	10	IV	Q7Dx2	2
4	3	POL-EPI (Dose Level 1)	12.5	10	IV	Q7Dx2	2
5	3	POL-EPI (Dose Level 2)	20	10	IV	Q7Dx2	2
6	3	POL-EPI (Dose Level 3)	30	10	IV	Q7Dx2	2

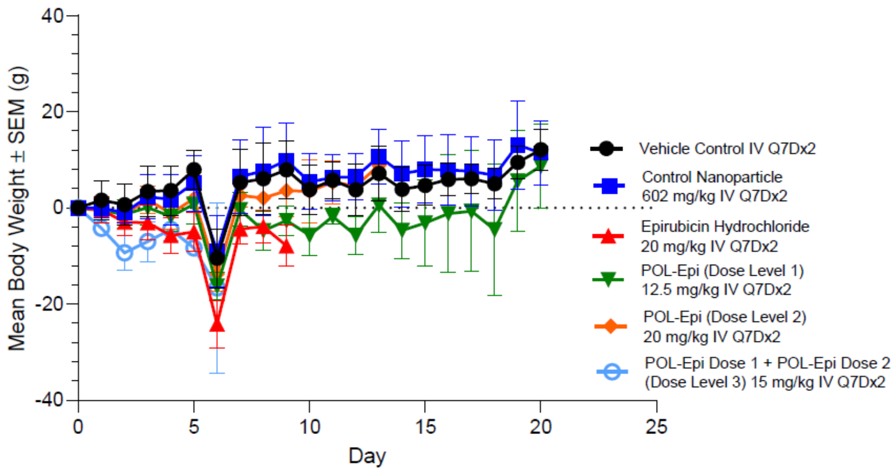

Figure S3. Mean percent body weight changes over time in non-tumor-bearing immunocompromised mice.

In tolerability study I, with non-tumor-bearing immunocompromised mice, Q7D×2 intravenous treatment of epirubicin hydrochloride at 20 mg/kg and POL-EPI at 20 mg/kg or 30 mg/kg were not tolerated in this study due to acute body weight loss resulting in many unscheduled deaths. The vehicle control, control nanoparticle at 602 mg/kg, and POL- EPI at 12.5 mg/kg were tolerated in this study. In the epirubicin hydrochloride 20 mg/kg group (Group 3), three animals were found dead on Day 10. Three animals in the POL-EPI (Dose Level 2) 20 mg/kg group (Group 5) were found dead two on Days 12 (2 animals) and 14 (1 animal). In the POL-EPI 30 mg/kg (Dose Level 3) group (Group 6), one animal was found dead on Day 6, and two animals were euthanized on Days 6. This tolerability study was repeated, and the dosing was revised as tolerability study II.

In tolerability study II, with non-tumor-bearing immunocompromised mice, Q7D×2 intravenous treatment of the vehicle control, epirubicin hydrochloride at 9 mg/kg, and POL-EPI at doses 9 mg/kg or 15 mg/kg were tolerated in this study.

Epirubicin hydrochloride at 12.5 mg/kg was not tolerated in this study. In the epirubicin hydrochloride 12.5 mg/kg (Dose Level 2) group (Group 3), one animal was euthanized on Day 15 due to severe body weight loss. Based on this study, the maximum tolerated dose identified for epirubicin hydrochloride was 9 mg/kg, and POL-EPI was 15 mg/kg.
